# Clinical phenotypes of obstructive sleep apnea: a cluster analysis based on sleep perception and sleep quality

**DOI:** 10.1007/s11325-023-02786-4

**Published:** 2023-02-28

**Authors:** Huasheng Wei, Jie Zhu, Fei Lei, Lian Luo, Ye Zhang, Rong Ren, Taomei Li, Lu Tan, Xiangdong Tang

**Affiliations:** 1 Sleep Medicine Center, Department of Respiratory and Critical Care Medicine, West China Hospital, Sichuan University, 28 Dian Xin Nan Jie, Chengdu, 610041 Sichuan China; 2 Department of Respiratory and Critical Care Medicine, Dazhou Central Hospital, Dazhou, China

**Keywords:** Cluster analysis, Obstructive sleep apnea, Sleep perception, Sleep quality

## Abstract

**Purpose:**

To determine obstructive sleep apnea (OSA) phenotypes using cluster analysis including variables of sleep perception and sleep quality and to further explore factors correlated with poor sleep quality in different clusters.

**Methods:**

This retrospective study included patients with OSA undergoing polysomnography (PSG) between December 2020 and April 2022. Two-step cluster analysis was performed to detect distinct clusters using sleep perception variables including discrepancy in total sleep time (TST), sleep onset latency (SOL), and wakefulness after sleep onset (WASO); objective TST, SOL, and WASO; and sleep quality. One-way analysis of variance or chi-squared tests were used to compare clinical and PSG characteristics between clusters. Binary logistic regression analyses were used to explore factors correlated with poor sleep quality.

**Results:**

A total of 1118 patients were included (81.6% men) with mean age ± SD 43.3 ± 13.1 years, Epworth sleepiness score, 5.7 ± 4.4, and insomnia severity index 3.0 ± 2.4. Five distinct OSA clusters were identified: cluster 1 (*n* = 254), underestimated TST; cluster 2 (*n* = 158), overestimated TST; cluster 3 (*n* = 169), overestimated SOL; cluster 4 (*n* = 155), normal sleep discrepancy and poor sleep quality; and cluster 5 (*n* = 382), normal sleep discrepancy and good sleep quality. Patients in cluster 2 were older, more commonly had hypertension, and had the lowest apnea–hypopnea index and oxygen desaturation index. Age and sleep efficiency were correlated with poor sleep quality in clusters 1, 2, and 5, and also AHI in cluster 2.

**Conclusion:**

Subgroups of patients with OSA have different patterns of sleep perception and quality that may help us to further understand the characteristics of sleep perception in OSA and provide clues for personalized treatment.

## Introduction

Obstructive sleep apnea (OSA) is a common sleep disorder characterized by repeated upper airway collapse during sleep resulting in apnea and hypopnea. OSA is associated with an increased risk of cardiovascular, neurocognitive, and metabolic comorbidities [1]. The diagnosis and management of OSA are currently based on the apnea–hypopnea index (AHI). However, OSA is highly heterogeneous, with variable clinical, polysomnographic, and pathophysiological manifestations [2], so the AHI cannot completely reflect all of these complex characteristics [3]. Cluster analysis is a powerful, data-driven approach used to identify and subclassify homogeneous subgroups of samples with similar characteristics within heterogeneous data, and the approach has also been applied to patients with OSA. Ye et al. first clustered patients with OSA based on symptoms and comorbidities. They found that the probabilities of having hypertension, diabetes, and cardiovascular disease were the highest in the minimally symptomatic group [4]. Kozu et al. divided patients with OSA into four clusters according to OSA severity, PaCO_2_, body mass index, and sleepiness, and found different serum C-reactive protein and leptin levels among clusters, which revealed different pathophysiological backgrounds of OSA [5]. A recent study identified nine clusters according to 10 clinically relevant and objectively identified comorbidities in patients with moderate-to-severe OSA, which found different cardiovascular risk, adherence to positive airway pressure, and long-term survival among clusters [6].

Sleep discrepancy, defined as the mismatch between subjective and objective sleep data, is an important clinical parameter in both insomniacs and patients with OSA [7]. The difference in self-reported and objective total sleep time (TST) is a widely used measure of discrepancy. Patients with OSA who underestimate their TST have been reported to have a propensity for objective insomnia, which might contribute to anxiety and depression caused by the perception of insufficient sleep [8]. Although the clinical significance of TST overestimation is uncertain, one study found that patients with OSA who overestimated TST were not necessarily those with less severe OSA [9]. In addition to TST discrepancy, subjective and objective differences in sleep onset latency (SOL) and wakefulness after sleep onset (WASO) have also been used as measures of sleep discrepancy. Sleep quality is also widely considered by clinicians, as it is closely related to physical and mental health, daytime function, and quality of life [10]. Previous studies of sleep quality in patients with OSA have reported that, in addition to intermittent hypoxia, impaired sleep quality can lead to microvascular damage [11, 12]. A recent study also reported that sleep quality mediates the relationship between the risk of OSA and acute stress in young adults [13].

Therefore, although different clustering algorithms have been used in patients with OSA and have included different variables including clinical symptoms, comorbidities, and adherence to positive airway pressure therapy [14], sleep discrepancy and sleep quality have not yet been included despite their potential clinical significance. Therefore, the purpose of this study was to include sleep perception and sleep quality as variables in clustering patients with OSA. Our findings contribute to the understanding of characteristics of sleep discrepancy in patients with OSA and may be helpful in the development of personalized OSA treatments.

## Methods

### Study design and setting

This was a retrospective study of patients with suspected OSA referred to the Sleep Medicine Center, West China Hospital of Sichuan University for polysomnography (PSG) between December 2020 and April 2022. The West China Hospital of Sichuan University Biomedical Research Ethics Committee approved the study (2022–898). Due to the retrospective design, the requirement for informed consent was waived. All data were anonymized and de-identified before release to the investigators.

### Participants

Participants over 18 years of age with the main symptoms of snoring, witnessed apnea, or daytime sleepiness and with polysomnography-derived AHI ≥ 5 events/h were included. Participants with an insomnia severity index (ISI) ≥ 8 and with other sleep disorders such as restless legs syndrome and REM sleep behavior disorder, neurological, or psychiatric disorders were excluded. Patients with other chronic comorbidities that may affect sleep perception and sleep quality, with a TST < 2 h measured by PSG, and with incomplete data were also excluded.

### Clinical evaluation and questionnaire

Patients underwent full clinical evaluation and had a complete medical history taken. Age, gender, weight, height, body mass index (BMI), smoking and alcohol use, OSA-related symptoms, and comorbidities were recorded. The Chinese version of the Epworth Sleepiness Scale (ESS) was used to assess the subjective daytime sleepiness [15]. The ISI questionnaire was used to assess the severity of insomnia, and a score ≥ 8 indicated clinical insomnia.

### Polysomnography (PSG)

A full-night PSG was conducted for each participant including continuous recordings from an electroencephalogram (F4–M1, C4–M1, O2–M1, F3–M2, C3–M2, O1–M2), electrooculogram (ROC-M1 and LOC-M2), electromyogram (submental and bilateral tibialis anterior), thermistors for nasal and oral airflows, nasal pressure swing, thoracic and abdominal excursion, finger pulse oximetry, electrocardiogram, and body position. Sleep and respiratory events were scored according to American Academy of Sleep Medicine guidelines (v2.3). Apnea was defined as a ≥ 90% decrease in airflow for ≥ 10 s, while hypopnea was defined as a ≥ 30% decrease in airflow for ≥ 10 s associated with an oxygen desaturation of ≥ 3% or an arousal. Total AHI was computed as the sum of apnea and hypopnea events divided by TST. The oxygen desaturation index (ODI) was the number of desaturations ≥ 3% divided by TST.

### Morning questionnaires

In the morning following overnight PSG, all participants were asked to complete a morning questionnaire that included four questions to assess subjective TST, SOL, WASO, and sleep quality. Subjective TST, SOL, and WASO were measured by open-ended questions and participants were asked to fill in a number. Sleep quality was measured on a Likert scale of “very good,” “good,” “poor,” and “very poor.”

### Subjective–objective sleep discrepancy

As previously, we used differences in subjective and objective TST, SOL, and WASO to reflect sleep discrepancy. A cutoff of 60 min was used for TST discrepancy (TSTdis), with TST underestimation defined as a TSTdis < − 60 min, normal perception as − 60 min ≤ TSTdis ≤ 60 min, and overestimation as TSTdis > 60 min [16]. A cutoff of 30 min was used for SOL discrepancy (SOLdis) [17], with SOL underestimation defined as SOLdis < − 30 min, normal perception as − 30 min ≤ SOLdis ≤ 30 min, and overestimation as TSTdis > 30 min. As no cutoff for WASO discrepancy (WASOdis) has been reported, this was considered as a continuous variable.

### Statistical analysis

TSTdis, SOLdis, WASOdis, objective TST (oTST), objective SOL (oSOL), objective WASO (oWASO), and sleep quality were used as variables in cluster analysis. As previously, TSTdis and SOLdis are presented as ordinal categorical variables and oSOL, oTST, oWASO, and WASOdis are presented as continuous variables. As the number of patients who rated sleep quality as “very good” and “very poor” were both small, these categories were combined with the “good” and “poor” categories and sleep quality was presented as a binary variable. There were no significant correlations or multicollinearity relationships between variables by Pearson correlation analysis and multicollinearity diagnosis. Moreover, the Kaiser–Meyer–Olkin (KMO) test for factor analysis suitability was < 0.6, indicating that the continuous variables were not suitable for principal component analysis. Therefore, we chose two-step cluster analysis to identify subgroups. After identifying clusters, continuous variables are presented as mean ± standard deviation (SD) and categorical variables as number (percentage). Differences between clusters were analyzed using one-way analysis of variance (ANOVA) or chi-squared tests. Post hoc Bonferroni correction analyses were conducted. Binary logistic regression analyses were performed to explore correlation factors for poor sleep quality in each cluster. SPSS v26.0 was used for all analyses (IBM Statistics, Armonk, NY, USA), and statistical significance was defined as *P* < 0.05.

## Results

**Fig. 1:**
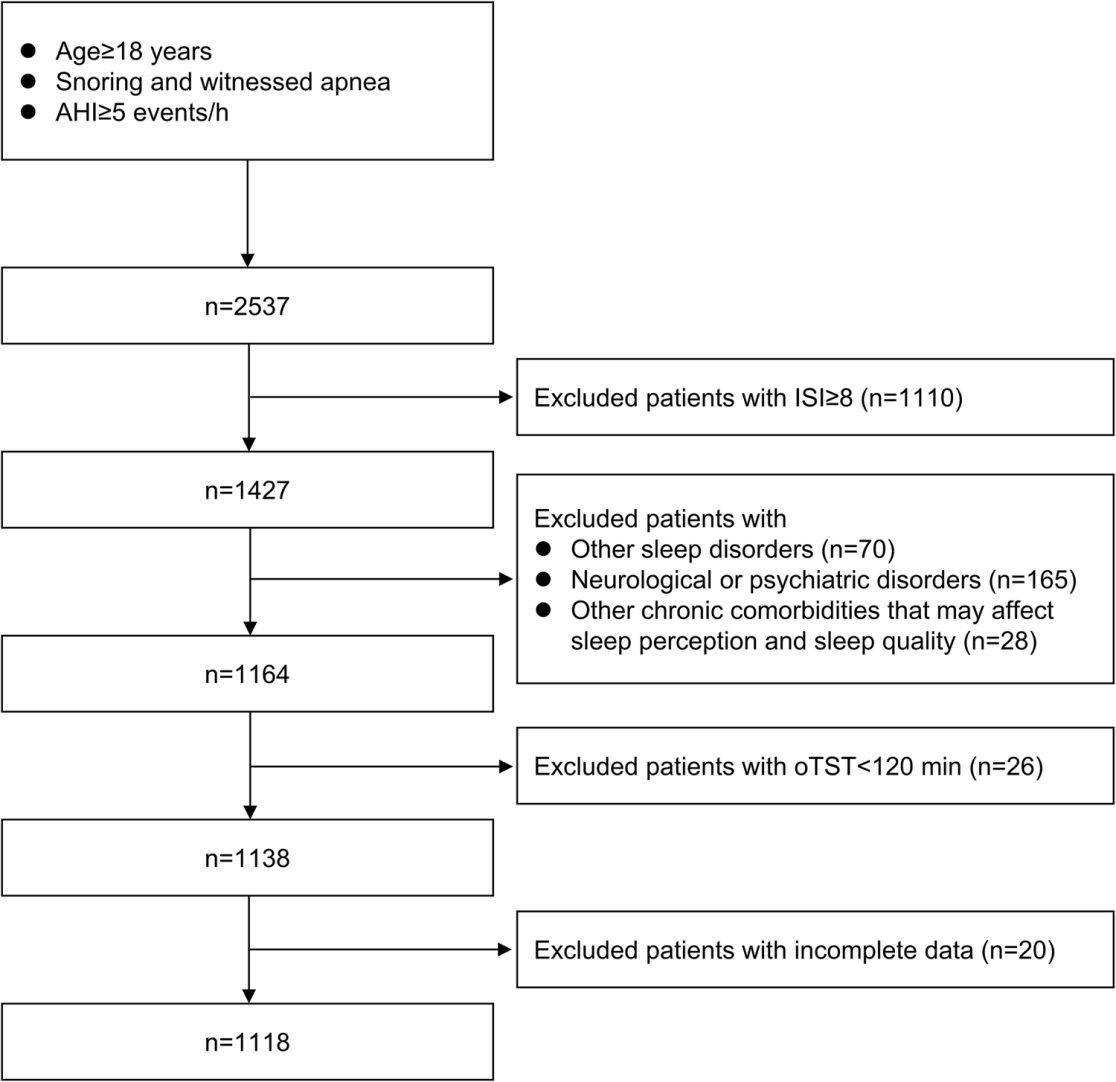
Patient flowchart. Abbreviations: AHI apnea- hypopnea index, ISI insomnia severity index, oTST objective total sleep time

**Fig. 2:**
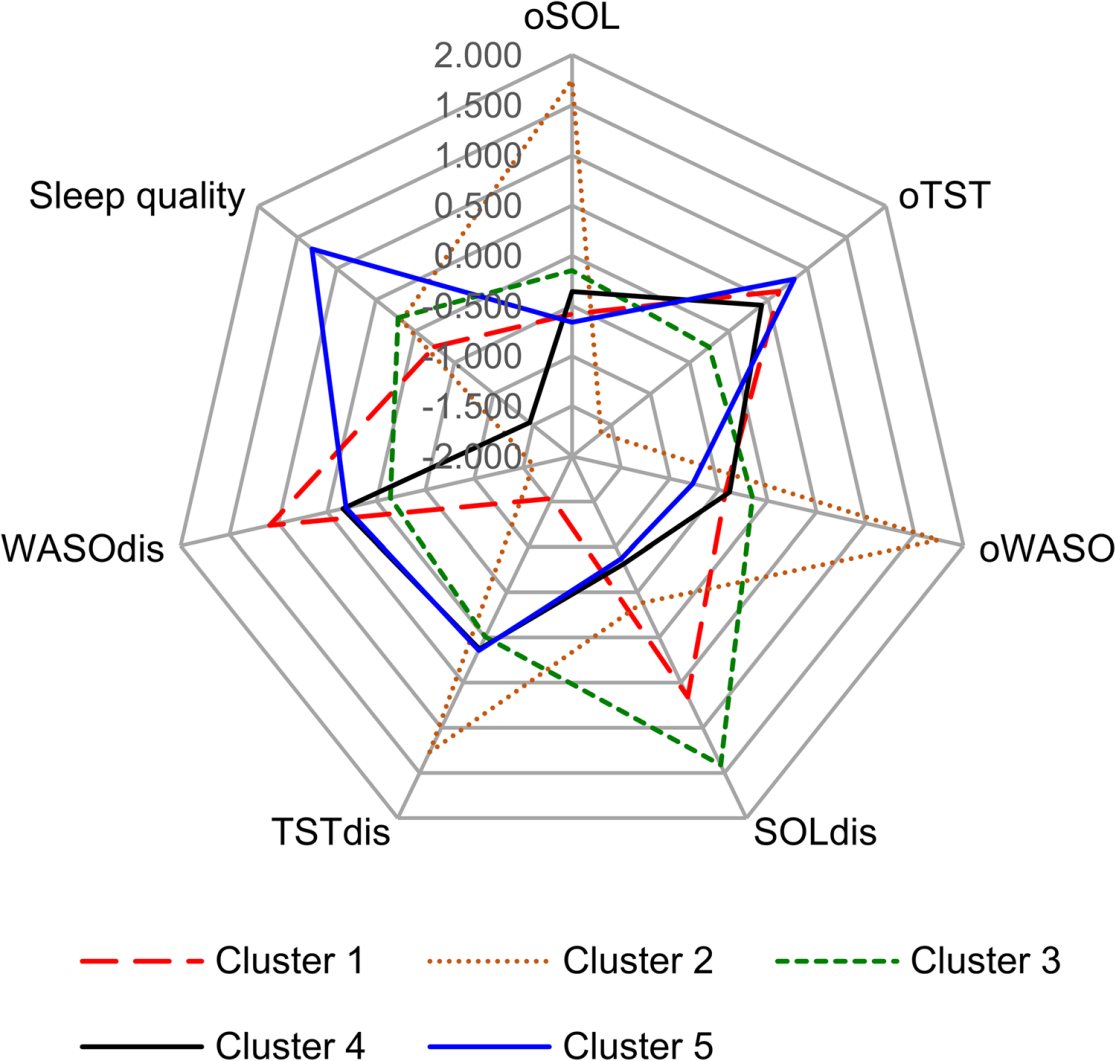
Radar plots of sleep perception and sleep quality profiles in the five clusters segregated by cluster analysis. Sleep perception parameters are shown as mean values, and sleep quality is shown as the percentage of good sleepers within the specified cluster. All variables were transformed into standardized Z-scores to standardize the scaling (mean 0, standard deviation 1) across the variables prior to plotting. Abbreviations: oTST objective total sleep time, oSOL objective sleep onset latency, oWASO objective wake after sleep onset, TSTdis total sleep time discrepancy, SOLdis sleep onset latency discrepancy, WASOdis wake after sleep onset discrepancy

A total of 2537 participants fulfilled the inclusion criteria, and 1118 participants were available for the final analysis. The patient flowchart is presented in Fig. 1. The mean age was 43.3 ± 13.1 years and the mean BMI was 26.1 ± 3.4 kg/m^2^, while the mean ESS and ISI scores were 5.7 ± 4.4 and 3.0 ± 2.4, respectively. Five distinct and highly discriminant clusters were identified (Fig. 2).

### Cluster profiles

- Cluster 1: Underestimated TST group. This group included 254 patients, 22.7% of the total sample. The majority of patients had an underestimated TSTdis, while 53.9% of patients had normal perception of SOLdis. Poor sleep quality was observed in 56.3% of patients.
- Cluster 2: Overestimated TST group. This group included 158 patients, 14.1% of the total sample. An overestimated TSTdis was present in 78.5% of patients, and 58.9% reported good sleep quality.
- Cluster 3: Overestimated SOL group. This group included 169 patients, 15.1% of the total sample. All patients overestimated their SOLdis, and 60.4% reported good sleep quality.
- Cluster 4: Normal sleep discrepancy and poor sleep quality group. This group included 155 patients, 13.9% of the total sample. Patients in this group had normal perception of TST and SOL but all reported poor sleep quality.
- Cluster 5: Normal sleep discrepancy and good sleep quality group. This group included 382 patients, 34.2% of the total sample. Patients in this group had normal perception of TST and SOL, and all reported good sleep quality.

### Demographic and clinical characteristics in clusters

The demographic and clinical characteristics according to cluster are presented in Table 1. Participants in cluster 2 were older than those in the other clusters, and the proportion of participants with hypertension was also highest in cluster 2. OSA-related symptoms were significantly different between clusters, but there were no significant differences in BMI, ESS score, ISI score, nor percentage of participants smoking, and using alcohol between the five clusters.

**Table 1:** Demographic and clinical characteristics of the participants in different clusters

Parameter	Cluster 1 Underestimated TST group *n* = 254	Cluster 2 Overestimated TST group *n* = 158	Cluster 3 Overestimated SOL group *n* = 169	Cluster 4 Normal sleep discrepancy and poor sleep quality group *n* = 155	Cluster 5 Normal sleep discrepancy and good sleep quality group *n* = 382	*P-value*
Gender, male (%)	214 (84.3%)	129 (81.7%)	134 (79.3%)	125 (80.7%)	310 (81.1%)	0.748
Age, years	42.9 ± 12.9	49.0 ± 14.9	41.1 ± 11.5	41.9 ± 12.2	42.6 ± 12.8	< 0.001
BMI, kg/m^2^	26.4 ± 3.5	26.1 ± 3.5	25.9 ± 3.2	25.8 ± 3.8	26.1 ± 3.4	0.500
ESS	5.9 ± 4.4	5.5 ± 4.5	5.1 ± 3.7	5.9 ± 4.4	5.9 ± 4.5	0.292
ESS > 10, *n* (%)	36 (14.2%)	26 (16.7%)	17 (10.1%)	18 (11.6%)	57 (14.9%)	0.408
ISI	3.2 ± 2.4	3.1 ± 2.4	2.9 ± 2.4	3.0 ± 2.5	2.9 ± 2.4	0.616
Smoker, *n* (%)	71 (28.0%)	57 (36.1%)	60 (35.5%)	46 (29.7%)	120 (31.4%)	0.345
Alcohol use, *n* (%)	86 (33.9%)	64 (40.5%)	52 (30.8%)	58 (37.4%)	129 (33.8%)	0.376
Snoring, *n* (%)	247 (97.2%)	149 (94.3%)	166 (98.2%)	147 (94.8%)	372 (97.4%)	0.163
Oppressive wake, *n* (%)	99 (39.0%)	67 (42.4%)	48 (28.4%)	66 (42.6%)	159 (41.6%)	0.031
Nocturia, *n* (%)	65 (25.6%)	51 (32.3%)	21 (12.4%)	24 (15.5%)	79 (20.7%)	< 0.001
Morning headache, *n* (%)	32 (12.6%)	23 (14.6%)	26 (15.4%)	32 (20.7%)	41 (10.7%)	0.042
Hypertension, *n* (%)	60 (23.6%)	55 (34.8%)	24 (14.2%)	33 (21.3%)	82 (21.5%)	< 0.001
CHD, *n* (%)	17 (6.7%)	6 (3.8%)	5 (3.0%)	2 (1.3%)	12 (3.1%)	0.053
Arrhythmia, *n* (%)	15 (5.9%)	11 (7.0%)	9 (5.3%)	5 (3.2%)	13 (3.4%)	0.296
CVD, *n* (%)	7 (2.8%)	13 (8.2%)	4 (2.4%)	6 (3.9%)	13 (3.4%)	0.037
Diabetes, *n* (%)	15 (5.9%)	13 (8.2%)	5 (3.0%)	9 (5.8%)	13 (3.4%)	0.101

*TST* total sleep time, *SOL* sleep onset latency, *BMI* body mass index, *CHD* coronary heart disease, *CVD* cerebrovascular disease, *ESS* Epworth sleepiness scale, *ISI* insomnia severity index

### Polysomnography characteristics in clusters

The polysomnography characteristics are presented in Table 2. The total AHI, AHI in non-rapid eye movement (NREM) sleep, and ODI were highest in cluster 1 and lowest in cluster 2. This was consistent with the observation of the lowest mean oxygen saturation in cluster 1 and no significant difference in nadir oxygen saturation between clusters. With respect to sleep structure, the percentage of NREM 1 was highest and NREM 2 lowest in cluster 1. The percentage REM sleep and sleep efficiency was highest in cluster 5.

**Table 2:** Polysomnographic characteristics of the participants in different clusters

Parameter	Cluster 1 Underestimated TST group *n* = 254	Cluster 2 Overestimated TST group *n* = 158	Cluster 3 Overestimated SOL group *n* = 169	Cluster 4 Normal sleep discrepancy and poor sleep quality group *n* = 155	Cluster 5 Normal sleep discrepancy and good sleep quality group *n* = 382	*P-value*
N1%TST, %	36.4 ± 19.1	34.5 ± 19.3	30.2 ± 16.7	32.3 ± 18.5	32.0 ± 18.5	0.006
N2%TST, %	45.8 ± 16.6	48.2 ± 16.6	50.6 ± 15.1	49.2 ± 15.8	48.6 ± 16.7	0.041
N3%TST, %	1.3 ± 2.9	1.3 ± 3.7	1.3 ± 2.9	1.6 ± 3.4	1.5 ± 3.2	0.784
R%TST, %	16.6 ± 5.4	16.1 ± 6.3	17.9 ± 5.3	16.9 ± 5.6	18.0 ± 5.3	< 0.001
SE, %	86.5 ± 9.3	68.0 ± 13.4	83.3 ± 10.1	85.6 ± 8.9	88.8 ± 7.2	< 0.001
ArI, events/h	33.6 ± 20.2	27.8 ± 16.3	28.7 ± 16.6	31.8 ± 22.3	31.1 ± 19.4	0.020
AHI (REM), events/h	47.9 ± 23.0	41.5 ± 21.6	42.6 ± 23.2	45.3 ± 24.3	43.9 ± 22.7	0.041
AHI (NREM), events/h	48.8 ± 18.5	38.0 ± 18.5	40.5 ± 18.5	44.1 ± 18.5	43.1 ± 18.5	0.001
AHI, events/h	49.8 ± 25.9	40.2 ± 24.8	42.0 ± 23.9	45.4 ± 28.6	44.2 ± 27.1	0.003
ODI, events/h	48.2 ± 28.3	37.4 ± 26.8	39.4 ± 25.5	42.2 ± 30.8	41.7 ± 29.7	0.002
Mean SaO_2_, %	92.3 ± 2.7	93.0 ± 2.1	93.1 ± 2.4	92.8 ± 2.6	92.4 ± 3.6	0.008
Nadir SaO_2_, %	74.0 ± 12.3	76.4 ± 12.6	76.6 ± 10.6	76.1 ± 13.2	74.4 ± 13.8	0.085
Mean OA duration, s	25.8 ± 9.7	23.9 ± 9.8	23.7 ± 9.9	24.2 ± 10.2	25.6 ± 9.6	0.061
Mean HR (REM), bmp	67.2 ± 15.5	64.7 ± 15.6	65.6 ± 14.7	65.8 ± 14.2	64.6 ± 15.4	0.317
Mean HR (NREM), bmp	66.0 ± 15.2	63.2 ± 14.6	64.0 ± 13.7	64.2 ± 14.4	63.5 ± 14.3	0.211

*TST* total sleep time, *SOL* sleep onset latency; N1%TST, N2%TST, N3%TST, and R%TST, the percentage of N1 period, N2 period, N3 period and R period in total sleep time; *SE* sleep efficiency, *ArI* arousal index, *AHI* apnea–hypopnea index, *AHI (REM)* AHI in rapid eye movement sleep, *AHI (NREM)* AHI in non-rapid eye movement sleep, *ODI* oxygen desaturation index, *SaO*_*2*_ oxygen saturation, *OA* obstructive apnea, *HR* heart rate, *bmp* beat per minute

### Sleep perception and sleep quality in clusters

Objective and subjective parameters, sleep discrepancy, and sleep quality are presented in Table 3. Objective TST was the shortest in cluster 2 and longest in cluster 5, while subjective TST was shortest in cluster 1 and longest in cluster 5. This resulted in the greatest TST underestimation in cluster 1 and greatest TST overestimation in cluster 2. With respect to objective SOL, this was longest in cluster 2 and shortest in cluster 5, while subjective SOL was longest in cluster 3 and shortest in cluster 5. All participants overestimated SOL and it was longest in cluster 3. Cluster 2 had the longest objective WASO, while cluster 5 had the shortest subjective WASO, with the greatest WASO underestimation observed in cluster 2. The percentage of good sleep quality ranged from 43.7 to 60.4%. Participants in cluster 5 all reported good sleep quality, while participants in cluster 4 all reported poor sleep quality.

**Table 3:** Sleep perception and sleep quality characteristics of participants in different clusters

Parameter	Cluster 1 Underestimated TST group *n* = 254	Cluster 2 Overestimated TST group *n* = 158	Cluster 3 Overestimated SOL group *n* = 169	Cluster 4 Normal sleep discrepancy and poor sleep quality group *n* = 155	Cluster 5 Normal sleep discrepancy and good sleep quality group *n* = 382	*P*-value
oTST, min	446.8 ± 53.2	338.5 ± 65.4	404.3 ± 50.6	436.2 ± 48.8	456.2 ± 39.1	< 0.001
sTST, min	329.1 ± 79.4	418.4 ± 76.5	394.2 ± 50.5	435.7 ± 46.0	456.7 ± 38.3	< 0.001
TSTdis, min	− 117.7 ± 53.9	79.9 ± 53.9	− 10.1 ± 29.6	− 0.5 ± 31.6	0.6 ± 28.8	< 0.001
oSOL, min	10.2 ± 10.1	32.3 ± 46.7	14.3 ± 15.9	12.3 ± 11.9	9.4 ± 10.2	< 0.001
sSOL, min	63.7 ± 67.1	55.2 ± 68.1	89.9 ± 54.0	22.7 ± 13.7	18.0 ± 12.3	< 0.001
SOLdis, min	53.5 ± 66.0	22.9 ± 64.3	75.6 ± 53.9	10.3 ± 12.7	8.6 ± 11.4	< 0.001
oWASO, min	60.0 ± 47.0	131.8 ± 73.3	69.1 ± 51.1	61.3 ± 44.3	48.9 ± 37.1	< 0.001
sWASO, min	67.5 ± 73.4	32.1 ± 36.9	27.5 ± 35.6	39.0 ± 36.1	25.3 ± 28.5	< 0.001
WASOdis, min	7.5 ± 75.3	− 99.7 ± 68.8	− 41.6 ± 50.3	− 22.3 ± 37.1	− 23.6 ± 33.6	< 0.001
Good sleep quality, *n* (%)	111 (43.7%)	93 (58.8%)	102 (60.4%)	0 (0.0%)	382 (100.0%)	< 0.001

*TST* total sleep time, *SOL* sleep onset latency, *oTST* objective total sleep time, *sTST* subjective total sleep time, *TSTdis* total sleep time discrepancy, *oSOL* objective sleep onset latency, *sSOL* subjective sleep onset latency, *SOLdis* sleep onset latency discrepancy, *oWASO* objective wakefulness after sleep onset, *sWASO* subjective wakefulness after sleep onset, *WASOdis* wakefulness after sleep onset discrepancy

### Factors correlated with poor sleep quality in each cluster

Age and sleep efficiency were negatively correlated with the risk of having poor sleep quality in clusters 1, 4, and 5. AHI was positively correlated with poor sleep quality in cluster 2, and sleep efficiency was the only factor that correlated with poor sleep quality in cluster 3 (Table 4).

**Table 4:** Factors associated with poor sleep quality among OSA patients

Good sleep versus poor sleep	Variable	*Β*	Standard error	Wald	*P*-value	OR (95% CI)
Cluster 1	Age	− 0.034	0.012	7.734	0.005	0.967 (0.944–0.990)
	SE	− 0.081	0.019	18.992	< 0.001	0.923 (0.890–0.957)
Cluster 2	Age	− 0.037	0.013	8.590	0.003	0.964 (0.940–0.988)
	AHI	0.074	0.030	6.322	0.012	1.077 (1.016–1.141)
	SE	− 0.074	0.018	9.234	0.002	0.958 (0.931–0.985)
Cluster 3	SE	− 0.726	0.328	16.334	< 0.001	0.929 (0.896–0.963)
Cluster 4 versus cluster 5	Age	− 0.017	0.008	4.305	0.038	0.983 (0.967–0.999)
	SE	− 0.058	0.013	20.583	< 0.001	0.944 (0.920–0.968)

*TST* total sleep time, *SOL* sleep onset latency, *OR* odds ratio, *CI* confidence interval, *SE* sleep efficiency, *AHI* apnea–hypopnea index

## Discussion

Here we present the first cluster analysis identifying OSA phenotypes using sleep perception and sleep quality. We defined five distinct phenotypes characterized by underestimated TST (cluster 1), overestimated TST (cluster 2), overestimated SOL (cluster 3), normal sleep discrepancy and poor sleep quality (cluster 4), and normal sleep discrepancy and good sleep quality (cluster 5). Cluster 2 contained older patients with a higher percentage of OSA-related symptoms and a history of hypertension. Although cluster 2 had the lowest objective sleep efficiency, patients in cluster 1 had more severe AHI, ODI, and mean oxygen saturation. Moreover, the factors correlated with poor sleep quality were different between clusters, with AHI positively and age and sleep efficiency negatively correlated with the risk of having poor sleep quality.

TST discrepancy has commonly been used to describe sleep perception [16]. In our study, the TST was overestimated and objective TST was the shortest in cluster 2, with a mean value of only 338.5 min. Moreover, patients in cluster 2 also had the lowest sleep efficiency and only 58.8% of participants reported good subjective sleep quality. Short objective TST is reported to be associated with an increased risk of cardiovascular disease in insomnia patients [18] and old adults with cognitive impairment [19]. In our previous study, we also found that short objective TST, rather than subjective TST, was independently associated with a risk of hypertension in patients with OSA [20]. Priou et al. also found that OSA severity and short objective TST were a cumulative association with the risk of hypertension [21]. Besides short TST, reduced sleep efficiency has also been correlated with increased risk of hypertension or other cardiovascular diseases [22]. In our study, we also found that the prevalence of hypertension and cerebrovascular diseases was highest in cluster 2. This implicated that more attention should be paid to OSA patients who reported good sleep but presenting short objective TST. In contrast to cluster 2, TST was underestimated in cluster 1, which had the shortest subjective TST but adequate objective TST and sleep efficiency, a phenomenon known as “negative sleep misperception” or “paradoxical insomnia” [16, 23]. As proposed by the attention-intention-effort model, a perception of sleeplessness can lead to active attempts to fall asleep, which could paradoxically contribute to the development of psychophysiological insomnia [24]. Rasskazova et al. also reported that high intention to sleep exacerbates sleep fragmentation, leading to worse sleep quality [25].

Both subjective SOL and SOLdis in cluster 3 were significantly longer than those in other clusters. This would be clinically apparent as subjective difficulty in falling asleep, although the majority of patients in this cluster (60.4%) rated sleep quality as good. Modest overestimation of SOL is common in both insomniacs and healthy sleepers [26], and this phenomenon might result from asynchronous thalamocortical deactivation while falling asleep in humans. Magnin et al. reported that during sleep onset the thalamus deactivates several minutes earlier than the cortex due to the influence of the sleep–wake functional circuits regulated by the hypothalamus and brainstem [27]. However, the overestimation of SOL in cluster 3 was significantly beyond the general range, revealing a specific OSA subtype. In addition, given that an overestimated SOL and underestimated TST are often observed in insomniacs [28], cluster 1 (underestimating TST) and cluster 3 (overestimating SOL) may represent two transitional or intermediate phenotypes of insomnia, though this now needs confirming in longitudinal studies. In addition, patients with OSA in these two clusters may have more potential to develop insomnia in the future and cognitive behavioral therapy for insomnia may help to improve objective TST and SOL that may be confirmed in the future study.

The objective WASO was longest and the WASOdis was shortest in cluster 2. Patients in cluster 2 may not correctly perceive wakefulness during sleep and misperceive it as sleep, leading to the perception of normal TST. Although no definite threshold for such wakefulness duration has been established, it was surprising to us that such a long WASO was underestimated, because short wakefulness seems to be more likely to be mistaken for sleep [26]. In addition, although it seems reasonable to speculate that the estimates of SOL, WASO, and TST are related, we found a predominance of SOL overestimation and WASO underestimation across all clusters regardless of TST perceptions. Similar results have been observed in other sleep disorders such as insomnia, narcolepsy, and restless legs syndrome [26, 28].

Our findings also suggested that the subjective sleep quality of patients with OSA could be different even when sleep perception was similar. Moreover, age was negatively associated with a risk of poor sleep in patients with OSA underestimating or overestimating TST. This is different to previous studies reporting that older age is associated with worse objective sleep quality [29]. There may be two reasons for this. The first may be due to the discrepancy in subjective and objective sleep quality; old adults with difficulty initiating or maintaining sleep may also report good subjective sleep quality [30]. The second may be the predominantly male population in our study. Unruh et al. reported that older age was less strongly associated with poorer subjective sleep quality in men than in women [31], which may be due to acclimatization to the deterioration in sleep quality over time. In addition, although AHI cannot adequately capture the heterogeneity of OSA, we found that AHI was positively associated with poor sleep in cluster 2, suggesting that AHI was a useful predictor of poor sleep quality in some specific OSA subgroups.

There are two strengths of our study. First, this was the first cluster analysis identifying OSA phenotypes using sleep perception and sleep quality and we use multiple indicators of sleep perception including SOL, TST, and WASO that make a comprehensive assessment of sleep perception in patients with OSA. Second, we excluded patients with comorbidity of insomnia and other sleep disorders, which eliminated the influence of these diseases on sleep perception and sleep quality.

This study has several limitations. First, we only recorded a single night of PSG data and night-to-night variability may have an effect on sleep perception. However, previous studies have demonstrated that night-to-night variability in sleep perception was mainly observed in older patients with insomnia [23, 30]. Second, due to the retrospective and single-center design, we could not determine whether the phenotypes in our study can generalize to a wider population. Third, as the participants in this study were relatively young, predominantly male, and few reported excessive daytime sleepiness, which may affect the generalizability of this study.

## Conclusion

In this cluster analysis, we obtained five clinical phenotypes of OSA with different sleep perception and sleep quality patterns. Our findings confirmed the heterogeneity and complexity of OSA and may contribute to a deeper understanding of sleep misperception in patients with OSA. The results may also provide clues for the development of more individualized treatment for OSA that will need to be confirmed in future studies.

## Data Availability

The datasets generated during and/or analyzed during the current study are available from the corresponding author on reasonable request.
